# 

**Published:** 1891-07-04

**Authors:** 


**PRICE ONE PENNY.**

No. 249, Vol. X.—July 4th, 1891.

[Registered at the General Post Office as a Newspaper for transmission in the United Kingdom and Abroad.]

WITH EXTRA NURSING SUPPLEMENT.

**Per Annum, 6s. 6d.; post free.**

**Monthly Parts, 6d.; post free 8d.**

**Ditto per Annum, 8s., post free.**

### THE HOSPITAL

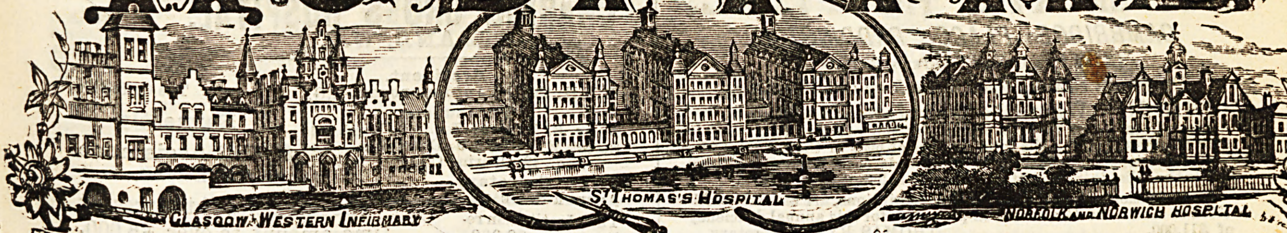

A WEEKLY JOURNAL OF

### Science, Medicine, Nursing and Philanthropy.

SATURDAY, JULY 4th, 1891.

**On the Side of the Commandments** . . . . . 157
**The History and Capabilities of Herbal Simples.** By W. T. Fernie, M.D.—XXXIV.—Fennel . . . . 158
**The Doctor's Armchair.**—A Disgraceful West-end Club . 159
**The University of Strasburg** . . . . . . . 160
**The Programme of the Hospital Saturday Fund** . 160
**Everybody's Page** . . . . . . . . . 161
**Notes and News** . . . . . . . . . 162
**General Charities** . . . . . . . . . 163
**Annotations.**—Doctors' Fees—Wanted, an English Haussmann—The Manchester Misadventure . . . . 164
**Scraps and Gleanings** . . . . . . . . . 163
**The Practitioners' Mirror and Retrospect—**
Medicine.—The Treatment of Whooping Cough, 165; The Source of the Bile Pigment—Pulsatilla . . . . 166
Surgery.—The Surgical Treatment of Purulent Pericarditis . . . . . . . . . . . . 166
**Recreation in Relation to the Health of the People**—V.—Recreation for the Body . . . . . . 168
**The Student of Medicine.**—Leading Athletes—Appointments—Vacancies—Pass Lists—Distribution of Prizes—Athletics.

### EXTRA SUPPLEMENT—THE NURSING MIRROR.

**En Passant.**—Queen's Nurses in Wales—A Jumble Sale—North and South—A Stockton Scandal—Short Items—Worcester Infirmary—The Second Thousand . . . lxxvii

**Lectures on Surgical Ward Work and Nursing.** By Alex. Miles, M.B. Edin., C.M., F.R.C.S.E.—Lecture XXVIII.—Rules for Bandaging . . . . . . lxxviii

**Everybody's Opinion.**—A Correction . . . . . lxxviii

**Appointment** . . . . . . . . . lxxviii

**Asylum Articles.** II.—The Asylum Day . . . . lxxix

**For Reading to the Sick—"Take Short Views"** . . lxxix

**A Plea for Help** . . . . . . . . . lxxx

**Wants and Workers** . . . . . . . . lxxx

**The Nurses' Wedding Gift** . . . . . . . lxxx

**The Lords' Committee** . . . . . . . . lxxxi

**Presentations** . . . . . . . . . lxxxi

**Notes and Queries** . . . . . . . . lxxxi

**Off Duty.**—Away from the Wards . . . . . lxxxii

**Amusements and Relaxation** . . . . . . lxxxii

